# KDM3A Attenuates Myocardial Ischemic and Reperfusion Injury by Ameliorating Cardiac Microvascular Endothelial Cell Pyroptosis

**DOI:** 10.1155/2022/4622520

**Published:** 2022-09-02

**Authors:** Bofang Zhang, Gen Liu, Bing Huang, Huafen Liu, Hong Jiang, Zheng Hu, Jing Chen

**Affiliations:** Department of Cardiology, Renmin Hospital of Wuhan University, Cardiovascular Research Institute, Wuhan University, Hubei Key Laboratory of Cardiology, Wuhan 430000, China

## Abstract

Cardiac microvascular endothelial cell ischemia-reperfusion (CMEC I/R) injury occurs in approximately 50% of acute myocardial infarction patients subjected to successful revascularization therapy. This injury leads to cardiac microcirculatory system dysfunctions, which seriously affect cardiac functions and long-term prognostic outcomes. Previously, we elucidated the role of lysine-specific demethylase 3A (KDM3A) in protecting cardiomyocytes from I/R injury; however, its roles in CMEC I/R injuries have yet to be fully established. In this study, hypoxia/reoxygenation (H/R) treatment significantly impaired CMEC functions and induced their pyroptosis, accompanied by KDM3A downregulation. Then, gain- and loss-of-function assays were performed to investigate the roles of KDM3A in CMEC H/R injury *in vitro*. KDM3A knockout enhanced CMEC malfunctions and accelerated the expressions of pyroptosis-associated proteins, such as NLRP3, cleaved-caspase-1, ASC, IL-1*β*, GSDMD-N, and IL-18. Conversely, KDM3A overexpression developed ameliorated alternations in CMEC H/R injury. *In vivo*, KDM3A knockout resulted in the deterioration of cardiac functions and decreased the no-reflow area as well as capillary density. Mechanistically, KDM3A activated the PI3K/Akt signaling pathway and ameliorated I/R-mediated CMEC pyroptosis. In conclusion, KDM3A is a promising treatment target for alleviating CMEC I/R injury.

## 1. Introduction

During the last decades, acute myocardial infarction (AMI) incidences have markedly declined as a result of the progressive implementation of preventive therapies and strict control of relative risk factors. However, AMI is among the main causes of mortality as well as morbidity worldwide [1]. Biologically, AMI results from abrupt occlusion of the coronary artery, leading to acute myocardial ischemia and irreversible damage to cardiomyocytes [2]. Therapeutic approaches that are aimed at ameliorating ischemic injuries have been integrated into clinical practice, and timely resumption of blood flow to ischemic myocardium has become a standard therapy [3, 4]. However, reperfusion may cause additional damage to the myocardium and compromise the therapeutic benefits, which is termed ischemic and reperfusion (I/R) injury [5]. This outcome exacerbates postinfarction maladaptive cardiac remolding and heart failure [6, 7]. The pathophysiology of I/R injury is multifactorial and is yet to be fully elucidated, and cardiac microcirculatory system dysfunction has emerged as a strong contributor.

As a highly structured organ, the heart consists of various cell types, such as cardiomyocytes, cardiac microvascular endothelial cells (CMECs), fibroblasts, and inflammatory cells [8]. This multicellularity enables intercellular communication within the heart, which then optimizes its functions [9]. Previous studies assessed the pathologic changes and associated mechanisms in cardiomyocytes during I/R injury. As the main component of the cardiac microcirculatory system, the specific contributions of CMECs to cardiomyocyte protection against I/R injury have drawn considerable attention. Indeed, CMECs are the most abundant cell types in the heart. Cardiomyocytes are surrounded by a capillary network that is pivotal for maintaining a sustained supply of oxygen and nutrients [10]. Effective revascularization of the infarct-associated artery does not imply sufficient perfusion of the ischemic myocardium, an outcome that is attributed to the myocardial “no-reflow” phenomenon (also known as microcirculatory I/R injury) [11]. Reperfusion induces swelling, deformation, and even death of CMECs, followed by distal embolization, microvascular plugging, and platelet aggregation, resulting in microvascular obstruction (MVO) [12]. The crosstalk between CMECs and cardiomyocytes plays a key role in regulating cardiac functions [13]. During I/R injury, insult to CMECs is the initial factor in cardiomyocyte damage [14, 15]. Therefore, elucidation of the molecular mechanisms involved in microcirculatory I/R injury will inform on maintenance of the normal functions of CMECs to treat myocardial I/R injury.

The complex pathophysiology of MVO is highly associated with inflammation and death of CMECs [16]. Pyroptosis is a unique form of inflammatory programmed cell death that is mediated in a NOD-like receptor thermal protein domain associated protein 3 (NLRP3) inflammasome/caspase-1-dependent manner [17]. Compared to apoptosis and necrosis, pyroptosis is characterized by rapid cell lysis, DNA damage, and massive release of intracellular contents, leading to violent proinflammatory responses [17–19]. Pyroptosis-associated cell death is significant in the myocardial I/R injury process [20, 21]. Besides, the NLRP3 inflammasome is a critical arbitrator of endothelial dysfunction-related inflammatory diseases, such as atherosclerosis, myocardial I/R injury, coronary arteritis, and ischemic stroke [22, 23]. Recently, Sun et al. reported that inhibition of the NLRP3/caspase-1 pathway ameliorates reperfusion injury-induced CMEC pyroptosis, thereby increasing capillary formations, mitigating inflammatory reactions, and attenuating infarct sizes [24]. Therefore, investigation of the key targets that regulate CMEC pyroptosis during I/R injury has therapeutic implications.

Lysine-specific demethylase 3A (KDM3A), which is also referred to as Jumonji domain-containing 1A (JMJD1A), is a member of the Jumonji c domain-containing protein family that functions as a histone demethylase to specifically demethylate the dimethylated histone H3 lysine 9 (H3K9me2) [25]. H3K9 methylation is deemed as a transcriptionally repressive mark; upregulation of KDM3A levels may inhibit H3K9me2 demethylation and activate the transcription of its specific target genes [26]. It has been reported that KDM3A has a role in various biological processes, such as cell proliferation, migration, tumor cell infiltration, and angiogenesis [27, 28]. It also has pathophysiological significance in diabetic vascular complications, AMI, and cardiac hypertrophy [29–31]. Therefore, KDM3A is pivotal in the regulatory network of cardiovascular diseases. In our latest study, KDM3A protected the myocardium from I/R injury and regulated inflammatory reactions as well as death of cardiomyocytes [26]. Nevertheless, the potential significance of KDM3A in CMEC I/R injury has not been conclusively established. We evaluated the roles of KDM3A in pathologic processes of CMEC I/R injury and elucidated on associated mechanisms.

## 2. Materials and Methods

### 2.1. Isolation of Primary CMECs and Establishment of Hypoxia/Reoxygenation Models *In Vitro*

Isolation of CMECs from wild-type (WT) and KDM3A global knockout rats (male, 20 days old) with SD backgrounds was done using the enzyme dissociation method as previously reported [26]. CMECs were cultured in an endothelial cell culture medium, and at a confluence of 80-90%, cells were subjected to anoxic and reoxygenation treatments. To simulate a hypoxic environment, an ischemic buffer (20 mM sodium lactate, 1 mM NaH_2_PO_4_, 0.5 mM sodium EDTA·2H_2_O, 24 mM NaHCO_3_, 118 mM NaCl, 2.5 mM CaCl_2_·2H_2_O, and 16 mM KCL) was supplemented to the culture medium. Incubation of cells was done in a trigas incubator that was filled with 5% CO_2_ and 95% N_2_ for 30 min. To mimic the reperfusion process, the ischemic buffer was replaced by a normal complete medium with further incubation for 6 h in a normoxic environment. To establish if the PI3K/Akt signaling pathway has a role in protective effects of KDM3A on CMECs, the specific PI3K/Akt signaling pathway inhibitor (LY294002 (No. HY-10108, MCE, 20 *μ*M)), was added 1 h before hypoxic and reoxygenation treatments.

### 2.2. Establishment of KDM3A Knockout Rats

The KDM3A global knockout rats were generated using the CRISPR/Cas9 gene-editing technology as previously described [26, 30]. We designed and created one single guide RNA (sgRNA) flanked exon 5 of the KDM3A gene in rats. After purifying and mixing the sgRNA and Cas9 mRNA, microinjections were performed on embryos. In order to determine the exact indel mutations of the generated founders, the PCR products were TA cloned and sequenced. Thereafter, the F1 heterozygote (KDM3A+/-) was obtained by mating the founder rat (the breeding line) with the WT (KDM3A+/+) SD rat strain. Screening of the KDM3A-KO strain was achieved via sibling mating of heterozygous F1 offspring.

### 2.3. Adenovirus Transduction

Adenoviral vectors encoding KDM3A (Ad-KDM3A) and control Ad-green fluorescent protein (GFP) (Ad-GFP) were constructed by GeneChem Co., Ltd. (Shanghai, China). At 50%-60% cell confluence, equal amounts of Ad-KDM3A or Ad-GFP at 50 MOI were dissolved into a serum-free medium and transfected for 4 h, respectively. Thereafter, the supernatant containing the adenovirus was abandoned and replaced with complete medium, followed by incubation for another 12 h. Successful transduction of the adenovirus was confirmed by the assessment of GFP expressions under fluorescent microscopy. The Ad-KDM3A-induced upregulation of the KDM3A protein was assessed by the Western blot assay.

### 2.4. Cell Proliferation and Migration Analyses

Proliferative abilities of CMECs in different groups were assessed by the cell counting kit-8 (CCK-8) assay. Cells were inoculated into 96-well plates (2 × 10^3^ cells/well), incubated, and supplemented with 20 *μ*L of the CCK-8 reagent (Dojindo Molecular Technologies, Inc., Japan) in every well followed by incubation for 4 h. Optical density (OD) value for all wells were obtained at 450 nm by the spectrophotometry system (Bio-Rad Laboratories, Inc., CA, USA).

The migration capacity of CMECs in each group was evaluated by the Transwell chamber (Corning Inc., Corning, NY, USA) and wound healing assays. Briefly, cells (1 × 10^5^) were inoculated into the upper chamber with serum-free medium (200 *μ*L) while 600 *μ*L medium with FBS (10%) was perfused into the lower chamber followed by 12 h of incubation. Nonmigrating cells on the upper side of the membrane were gently wiped off while cells that had migrated to the lower membrane surface were formaldehyde (4%)-fixed and stained with crystal violet (0.5%) in the dark. At least three wells from each group were selected, and one random field of each well was chosen to count the cells that were on the lower surface of the membrane by light microscopy (Olympus Corporation, Tokyo, Japan). Wound healing assays were performed using culture inserts (No. 81176, Cat., USA). In this assay, 70 *μ*L of CMEC suspension containing 1 × 10^4^ cells was inoculated into each side of the inserts and incubated for 12 h. The culture insert was gently removed when the cell layer reached confluency. One mL of endothelial cell medium was extracted to fill the dishes, after which the CMECs were incubated for another 12 h. Representative images were obtained by light microscopy, and the wound closure rate for each group was calculated using the ImageJ software.

### 2.5. Tube Formation and Sprouting Assay

After various treatments, angiogenesis abilities of CMECs were measured by tube formation and sprouting assays. Briefly, 80 *μ*L liquid Matrigel was infused into each well of 96-well plates and sat still for 1 h to solidify. Then, trypsinized CMECs were resuspended into the complete medium and seeded into Matrigel-coated wells (1 × 10^4^ cells/well). After 8 h, capillary-like tube structures were determined by light microscopy; total tube length and tube loops for each well in one high-power field were evaluated by ImageJ software. For the sprouting assay, trypsinized CMECs were resuspended in 0.2% methylcellulose and adjusted to 1000 cells/spheroid per 25 *μ*L. Using a multichannel pipette (25 *μ*L), droplets were placed on the cover of 15 mL plates, covered with the plate, flipped upright, and incubated overnight. 12 h later, 10 mL of PBS with 10% FBS was pipetted onto the cover plates to collect the spheroids that were centrifuged for 5 min at 300 g. A collagen matrix was used to resuspend the spheroids, after which 500 *μ*L was transferred to each well of the 24-well plate. Subsequently, 0.5 mL endothelial cell culture medium was added on top of the collagen matrix followed by incubation for another 12 h. At the end of the experiment, spheroids were imaged for quantification of sprout counts and determination of sprout length.

### 2.6. Cell Death Analysis

In vitro, CMEC vitality was assessed using Hoechst 33342/PI double fluorescent staining. Briefly, CMECs at a density of 5 × 10^5^ were seeded onto coverslips and subjected to corresponding treatments. Then, cells in different groups were incubated with Hoechst 33342 for 10 min and PI for 25 min away from light. Stained cells were observed by microscopy. The percentage of Hoechst 33342/PI dual-positive cells was measured to determine the apoptotic rate. To measure *in vivo* cell death ratios, TUNEL staining was performed as instructed by the manufacturer. The myocardium (red) was marked by the anti-*α*-actinin antibody, while DAPI and green fluorescent dye was used to label entire (blue) and apoptotic (green) nuclei, respectively. The apoptotic index (AI) was determined as the ratio of TUNEL-positive cells (green) to total cells (blue).

### 2.7. Secretory Capacity Assay

To measure the secretory capacity of CMECs in different groups *in vitro*, levels of Nitric Oxide (NO) in the cell culture supernatants were assayed by the enzyme-linked immunosorbent assay (ELISA; Nanjing Jiancheng Biology Engineering Institute, China) as instructed by the manufacturer. eNOS activities were determined by the conversion of L-[14C] arginine to L-[14C] citrulline in the cell lysate of each group (Cayman Chemical, MI).

### 2.8. Establishment of Rat I/R Injury Models and Assessment of Myocardial Infarct Sizes

The myocardial I/R model was established as reported [26]. Rats (male, 6-8 weeks, 220-250 g) were anaesthetized via intraperitoneal administration of pentobarbital sodium (40 mg/kg) (Sigma-Aldrich, USA), and a left parasternal incision was performed. Then, left anterior descending (LAD) ligation was performed for 30 min on rats, followed by reperfusion for 24 h. Sham-operated rats underwent the same operations, minus LAD occlusion or reperfusion.

Evans blue and TTC staining were done to detect myocardial infarct sizes. At 24 h after I/R injury, LAD was religated at the site of prior occlusion, and Evans blue (2.0%, 1 mL) (Sigma-Aldrich, USA) was administered into the aorta. Hearts from each rat group were collected, transected at the papillary muscle plane, and incubated with 1% TTC. White areas (negatively stained) denoted the infarcted myocardium. The proportion of myocardial infarct was determined as infarcted areas/total left ventricle (LV) areas × 100%.

### 2.9. Evaluation of Cardiac Functions

An echocardiography assay was conducted to assess cardiac LV functions at baseline and at 24 h after reperfusion. Ultrasound images were obtained from the parasternal short axis at the midpapillary muscle level, after which left ventricular fractional shortening (LVFS) and left ventricular ejection fraction (LVEF) were subsequently calculated.

### 2.10. Determination of No-Reflow Area and Capillary Density

The no-reflow area of each group after I/R treatment was measured by thioflavin-S and Evan's blue dual-staining. At 24 h after reperfusion, thioflavin-S (4%) was administered via the inferior vena cava and stained for 5 min. Then, LAD was religatured at the same occlusion location, and 3% Evan's blue was administered to the nonschemic myocardium via the jugular vein for staining. Thereafter, rat hearts were obtained from every group, transected at the papillary muscle plane, and exposed to ultraviolet light. Myocardium tissues with blue fluorescence indicated normal perfusion. Areas with no-reflow (attenuated or absent thioflavin-S fluorescence) were determined by the software of ImageJ and presented as a ratio of LV.

To investigate capillary densities of different groups, representative sections of different groups were stained with the CD31 antibody as previously described [32].

### 2.11. Western Blot Assay

In this assay, CMECs and myocardial tissue samples were lysed using the RIPA lysis buffer for protein extraction as instructed by the manufacturer. Then, 4.5 *μ*g/*μ*L total proteins from each group were separated by 10% SDS-PAGE, transferred onto polyvinylidene fluoride membranes (Millipore, MA, USA), blocked using 5% nonfat dried milk, and incubated overnight with primary antibodies against KDM3A, p-Akt, NLRP3, ASC, IL-1*β*, GSDMD-N, cleaved-caspase-1, and IL-18. Further incubation was performed with appropriate secondary antibodies. An enhanced chemiluminescence system (Thermo Fisher Scientific, Inc.) was used to visualize the protein bands. GAPDH was the internal control. BandScan 4.30 (Glyko, Novato, CA, USA) was used to semiquantify the blots.

### 2.12. Statistical Analysis

Continuous variables are presented as the mean ± SD. Student's *t*-test was used for between-group comparisons of means while one-way analysis of variance (ANOVA) was used for among-group comparisons, followed by the Student-Newman-Keuls (SNK) *q* tests for pairwise comparisons. *p* ≤ 0.05 denoted significance. The SPSS 19.0 and GraphPad Prism (Version 8.2.1) software were used for analyses.

## 3. Results

### 3.1. H/R Treatment Induced CMEC Injury and Dysfunction

**Figure 1:**
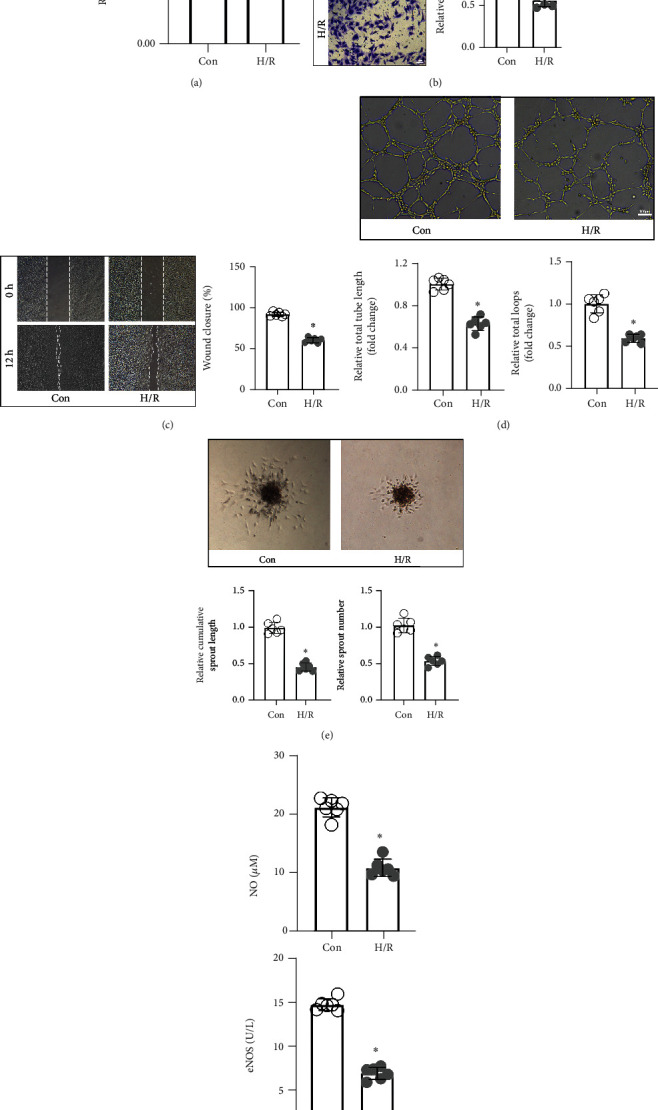
H/R treatment resulted in CMEC injury and dysfunction. (a) The proliferative capacity of CMECs was assessed by the CCK-8 assay, and the OD value was the quantitative indicator (*n* = 6, ^∗^*p* < 0.05, relative to the Con group). The migration capacity of CMECs was measured by the transwell chamber assay (b) and wound healing assay (c), respectively. The number of cells on the lower membrane surface (scale bar = 50 *μ*m) and wound closure rate of each group were calculated (*n* = 6,  ^∗^*p* < 0.05, relative to the Con group). The neovascularization capacity of CMECs was measured by tube formation (d) and sprouting assays (e). Total tube length and total loops (scale bar = 50 *μ*m), as well as cumulative sprouts and sprout counts for each group, were evaluated (*n* = 6,  ^∗^*p* < 0.05, relative to the Con group). (f) To detect the synthesis and secretion capacities of CMECs, eNOS and NO levels in supernatants of every group were determined (*n* = 6, ^∗^*p* < 0.05, relative to the Con group).

The capacities of CMECs for proliferation, migration, angiopoiesis, and secretion were determined before and after H/R treatment.  Figure 1(a) shows that cell proliferative potential in the H/R group was markedly reduced, relative to the control group. Findings from the transwell chamber assay indicated that there were fewer cells that had migrated to the lower membrane surface in the H/R group (Figure 1(b)). The wound healing rate of the H/R group was also reduced, relative to the control group (Figure 1(c)). Migration capacities of CMECs were impaired after H/R treatment. Neovascularization is a vital ability of endothelial cells. In Figure 1(d), total tube length and total vascular loop number of CMECs in the H/R group were less than those of the control group. Moreover, sprout numbers and sprout length of CMECs were decreased after H/R treatment (Figure 1(e)). Then, eNOS and NO levels in the supernatant were measured to assess cell synthesis and secretion capacities for each group. It was found that eNOS and NO levels were markedly suppressed in the H/R group (Figure 1(f)). As a result, H/R treatment led to CMEC injury and cellular dysfunction.

### 3.2. H/R Treatment Induced CMEC Pyroptosis and Altered KDM3A Expressions

**Figure 2:**
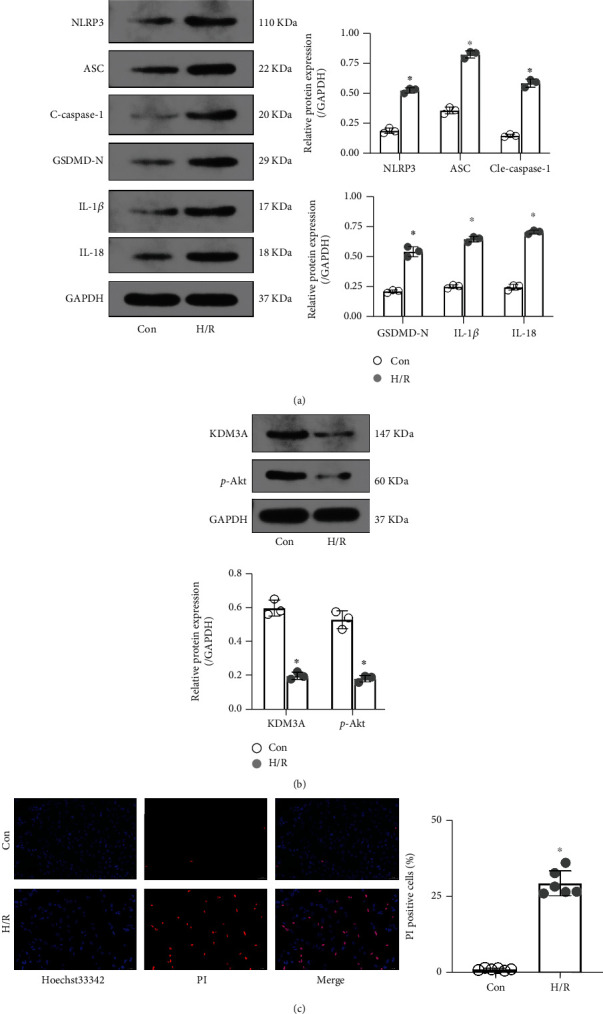
H/R treatment induced CMEC pyroptosis accompanied by downregulation of KDM3A and p-Akt. (a) The Western blot assay was conducted to evaluate the levels of pyroptosis-related proteins, including NLRP3, GSDMD-N, cleaved-caspase-1, ASC, IL-1*β*, and IL-18 (*n* = 3,  ^∗^*p* < 0.05, relative to the Con group). (b) The expressions of KDM3A and p-Akt before and after H/R treatments were also detected by Western blot assay (*n* = 3,  ^∗^*p* < 0.05, relative to the Con group). (c) Hoechst 33342/PI double fluorescent staining was performed to detect cell death *in vitro*, and the percentage of PI-positive cells was calculated in each group (*n* = 6,  ^∗^*p* < 0.05, relative to the Con group).

NLRP3/caspase-1-dependent pyroptosis is positively associated with I/R injury [20, 21, 24]. To determine if pyroptosis was induced in CMECs during H/R, key pyroptosis-associated indicators, such as NLRP3, ASC, cleaved-caspase-1, GSDMD-N, IL-1*β*, and IL-18, were detected by Western blotting. In Figure 2(a), expressions of the above indicators were significantly enhanced in response to H/R treatment, relative to the control group. There was an obvious increase in the number of Hoechst 3342/PI dual-stained positive cells in the H/R-treated group. However, as a crucial mediator in cardiovascular diseases, expressions of KDM3A and its downstream target (p-Akt) were downregulated after H/R treatment. These findings imply that CMEC pyroptosis is involved in H/R and KDM3A is an important contributor during the process.

### 3.3. KDM3A Knockout Exacerbated H/R-Induced CMEC Injury and Dysfunction

**Figure 3:**
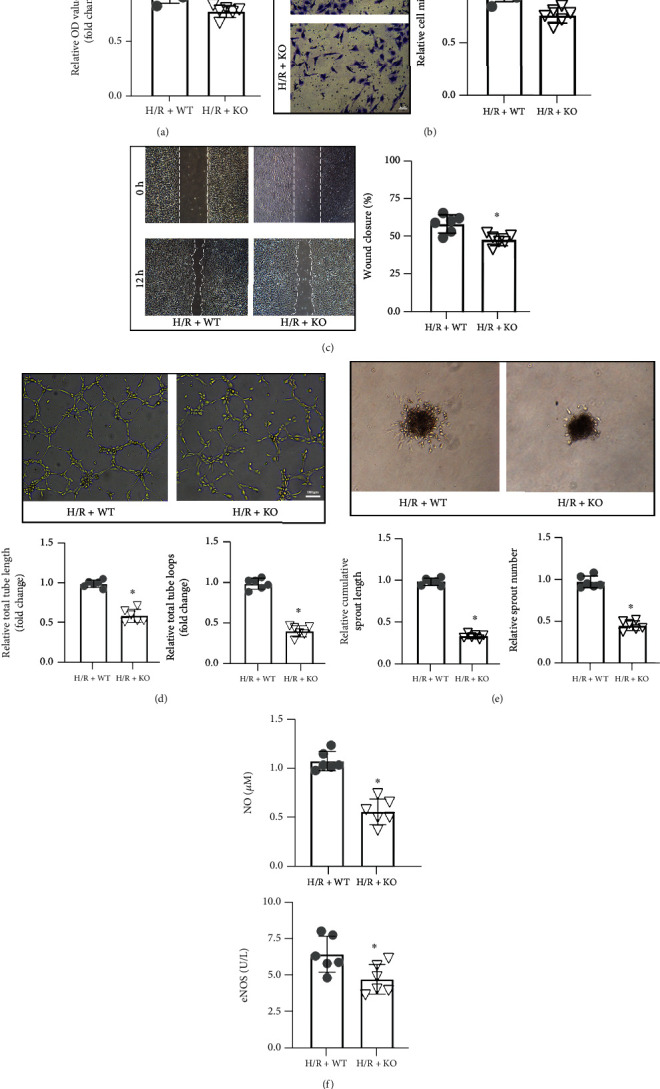
KDM3A knockout exaggerated H/R-induced CMEC injury and dysfunction. (a) The CCK-8 assay was used to estimate the proliferative abilities of WT CMECs and KDM3A-KO CMECs during H/R (*n* = 6,  ^∗^*p* < 0.05, compared to the H/R+WT group). (b, c) Transwell chamber and wound healing assays were conducted to assess the migration capacities of CMECs before and after KDM3A knockout during H/R (*n* = 6,  ^∗^*p* < 0.05 relative to the H/R+WT group). (d, e) Tube formation and sprouting assays were performed to investigate the neovascularization capacities of CMECs in the H/R+WT and H/R+KO groups (*n* = 6,  ^∗^*p* < 0.05, relative to the H/R+WT group). (f) Levels of eNOS and NO in supernatants were measured to assess the synthesis and secretion capacities of CMECs in every group after H/R treatment (*n* = 6,  ^∗^*p* < 0.05, relative to the H/R+WT group).

To establish the effects of KDM3A on CMECs after H/R treatment, loss-of-function assays were conducted. The KDM3A-KO rat strain was established, and KDM3A gene deleted CMECs were isolated. Determination of whether KDM3A gene knockout affected CMEC functions under normoxic conditions revealed that there were no marked differences between the WT and KDM3A-KO groups with regard to their capacities for cell proliferation, migration, angiogenesis, and secretion (Supplementary Figure 1). Thereafter, the cellular functions were measured again after cells had been subjected to H/R treatments. In Figure 3(a), proliferation capacities of cells in the KDM3A-KO+H/R group were markedly weakened, relative to the WT+H/R group. Besides, H/R markedly inhibited the migration capacities of CMECs, which were exacerbated by KDM3A knockout (Figures 3(b) and 3(c)). Data from tube formation and sprouting assays showed that KDM3A knockout impaired the angiogenic potency of CMECs, as manifested by reduced tube length and total tube loop number as well as the reduced sprout numbers and sprout length in the KDM3A-KO+H/R group (Figures 3(d) and 3(e)). Regarding cell secretion functions, Figure 3(f) shows that KDM3A gene deficiency inhibited the secretion of protective cytokines. These findings imply that KDM3A knockout aggravates H/R-induced CMEC malfunctions.

### 3.4. KDM3A Knockout Deteriorates H/R-Induced CMEC Pyroptosis and Suppressed p-Akt Levels

**Figure 4:**
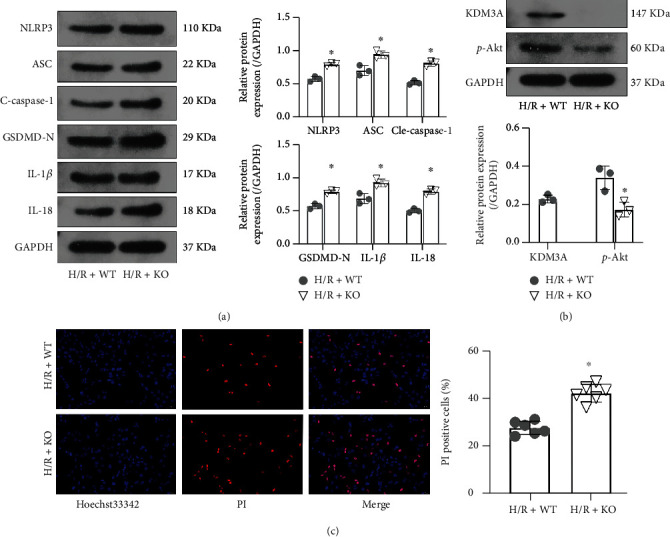
KDM3A knockout promoted H/R-induced CMEC pyroptosis and suppressed p-Akt levels. (A) Western blotting was performed to determine the levels of pyroptosis-related proteins, such as NLRP3, GSDMD-N, cleaved-caspase-1, ASC, IL-1*β*, and IL-18 (*n* = 3,  ^∗^*p* < 0.05 relative to the H/R+WT group). (b) Western blotting was also performed to verify the effects of KDM3A knockout and to detect p-Akt levels after KDM3A knockout during H/R stimulation (*n* = 3,  ^∗^*p* < 0.05, relative to H/R+WT group). (c) CMECs that had lost their vitality in the H/R+WT and H/R+KO groups were analyzed by Hoechst 33342/PI double fluorescent staining (*n* = 6,  ^∗^*p* < 0.05, compared to the H/R+WT group).

To establish whether KDM3A can regulate CMEC pyroptosis during H/R-mediated injury and to investigate the underlying mechanisms, we assessed the levels of pyroptosis-associated proteins and p-Akt levels before and after KDM3A knockout. In Figure 4(a), KDM3A knockout upregulated NLRP3, cleaved-caspase-1, ASC, GSDMD-N, IL-1*β*, and IL-18 levels. Moreover, the increase in the number of Hoechst 3342/PI dual-staining positive cells implies that KDM3A knockout promoted cell death. However, p-Akt protein levels were markedly decreased after KDM3A gene deletion, suggesting that KDM3A knockout exacerbated H/R-induced CMEC pyroptosis by suppressing the PI3K/Akt signaling pathway.

### 3.5. KDM3A Overexpression Ameliorated H/R-Induced CMEC Injury and Dysfunction, while LY294002 Treatment Reversed These Effects

**Figure 5:**
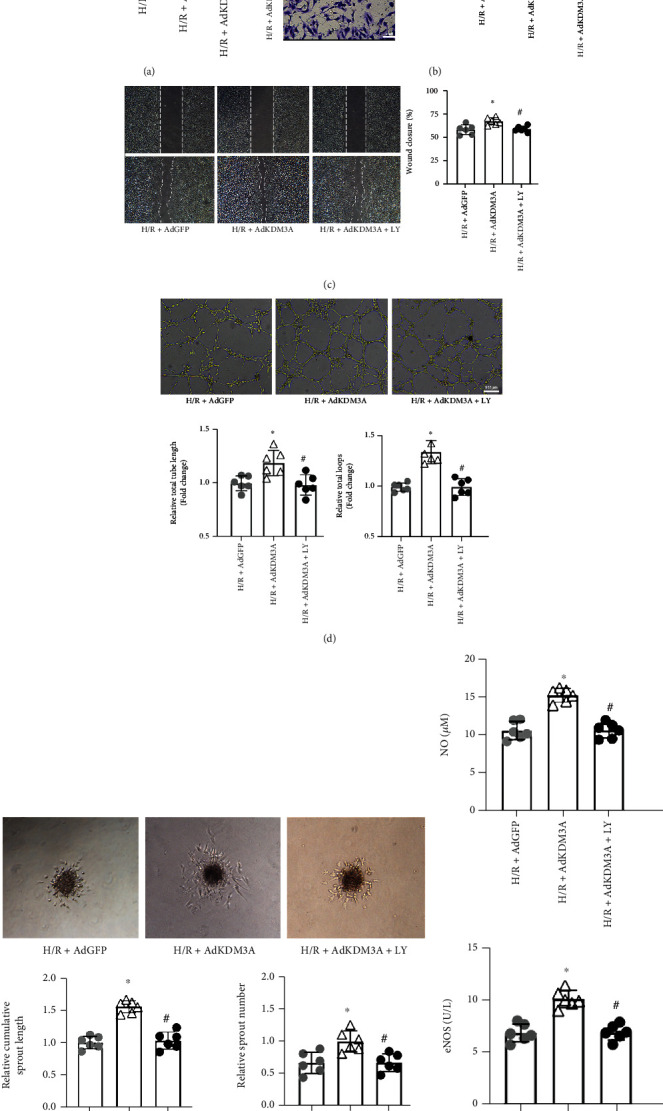
KDM3A overexpression ameliorated H/R-induced CMEC injury and dysfunctions, while suppression of the PI3K/Akt signaling pathway reversed these beneficial effects. (a) The CCK-8 assay was conducted to measure the proliferative abilities of CMECs in each group (*n* = 6,  ^∗^*p* < 0.05, relative to the H/R+Ad-GFP group, ^#^*p* < 0.05, compared to the H/R+Ad-KDM3A group). (b, c) Migration abilities of CMECs after KDM3A overexpression or/and PI3K/Akt inhibition were detected by transwell chamber and wound healing assays (*n* = 6,  ^∗^*p* < 0.05, compared to the H/R+Ad-GFP group, ^#^*p* < 0.05, compared to the H/R+Ad-KDM3A group). (d, e) Images of tube formation and sprouting assay for each group. Neovascularization capacities of CMECs after KDM3A overexpression or/and PI3K/Akt signaling pathway inhibition were evaluated. (f) eNOS and NO levels in supernatants of each group were measured to determine the synthesis and secretion capacities of CMECs (*n* = 6,  ^∗^*p* < 0.05, compared to the H/R+Ad-GFP group; ^#^*p* < 0.05, compared to the H/R+Ad-KDM3A group).

To validate the putative function of KDM3A, gain-of-function experiments were also performed. We tried to construct CMECs-specific KDM3A overexpression rats; unfortunately, the embryo does not survive. As a result, Ad-KDM3A recombinant adenovirus vector was constructed as an alternative and transfected into CMECs to upregulate KDM3A protein levels. Proliferative capacities of CMECs in different groups were evaluated by the CCK-8 assay. In Figure 5(a), KDM3A overexpression attenuated the H/R-induced impairment of proliferation capacities. However, pretreatment with LY294002, a specific PI3K/Akt signaling pathway inhibitor, ameliorated the advantage of KDM3A upregulation. Similarly, KDM3A overexpression improved the migration capacity of CMECs after H/R treatment, as manifested by the migration of more cells to the lower membrane surface and enhanced wound healing rate in the Ad-KDM3A+H/R group. LY294002 treatment ameliorated these beneficial effects (Figures 5(b) and 5(c)). Consistently, to a large extent, Ad-KDM3A transfection compensated for the adverse effects of hypoxia on angiogenic functions of CMECs. Tube length and total tube loop number as well as sprout numbers and sprout length in the Ad-KDM3A+H/R group were markedly increased, relative to the Ad-GFP+H/R group. Variations of these parameters were reversed by suppression of the PI3K/Akt signaling pathway (Figures 5(d) and 5(e)). Regarding cell synthesis and secretion functions, Figure 5(f) shows that overexpressed KDM3A significantly enhances the secretion of eNOS and NO in response to H/R, whereas LY294002 eliminates these beneficial effects. Collectively, these findings show that KDM3A has significant roles in the repair of impaired CMECs' functions. Its multiple protective effects against H/R might be associated with the PI3K/Akt signaling pathway.

### 3.6. KDM3A Overexpression Mitigated H/R-Induced CMEC Pyroptosis via PI3K/Akt Signaling Pathway Activation

**Figure 6:**
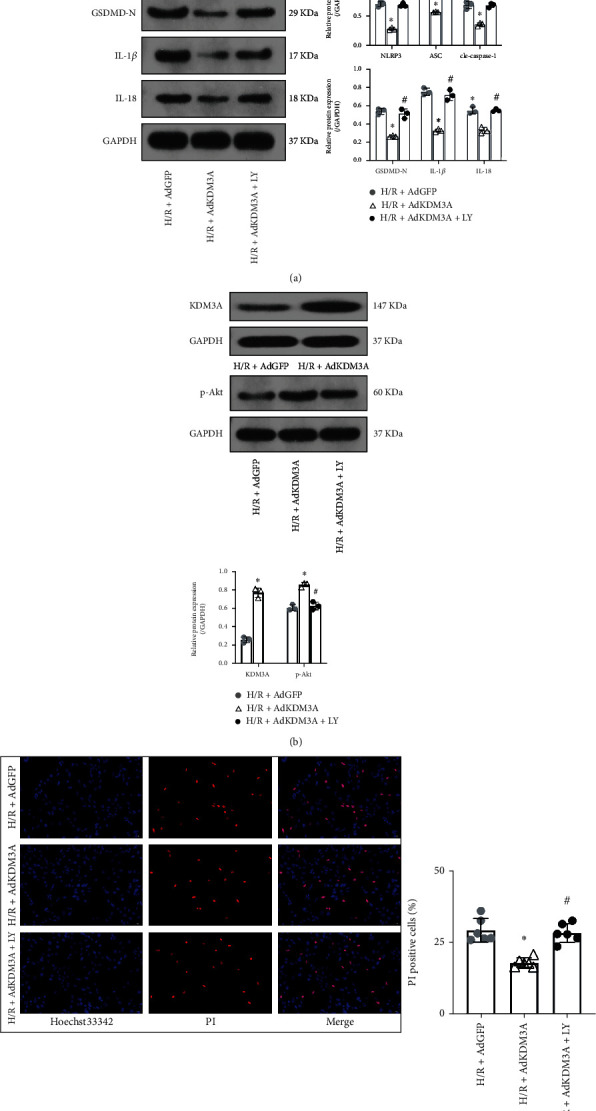
KDM3A overexpression mitigated H/R-induced CMEC pyroptosis through activating PI3K/Akt signaling pathway. (a) Protein levels of NLRP3, ASC, GSDMD-N, cleaved-caspase-1, IL-1*β*, and IL-18 were analyzed by Western blot assay. KDM3A overexpression significantly downregulated the expressions of pyroptosis-associated proteins while LY294002 treatment ameliorated these beneficial effects in the setting of H/R (*n* = 3,  ^∗^*p* < 0.05, compared to the H/R+Ad-GFP group, ^#^*p* < 0.05, compared to the H/R+Ad-KDM3A group). (b) Western blot assay was also performed to verify the transfection effects of Ad-KDM3A and to detect the expressions of p-Akt after KDM3A overexpression or/and PI3K/Akt inhibition under the condition of H/R stimulation. (c) Hoechst 33342/PI double fluorescent staining was conducted to assess cell death in each group (*n* = 6,  ^∗^*p* < 0.05, compared to the H/R+Ad-GFP group, ^#^*p* < 0.05, compared to the H/R+Ad-KDM3A group).

Given that KDM3A deletion aggravated H/R-induced CMEC pyroptosis, we evaluated the effects of overexpressed KDM3A. In Figure 6(a), Ad-KDM3A transfections markedly inhibited the H/R-induced upregulation of pyroptosis-associated proteins. PI3K/Akt signaling pathway suppression by LY294002 ameliorated these beneficial effects. Consistent with these results, KDM3A upregulation markedly reduced H/R injury-induced cell death, while LY294002 pretreatment reversed these outcomes. Meanwhile, treatment with LY294002 suppressed KDM3A overexpression-induced PI3K/Akt signaling pathway activation. Evidence from the gain- and loss-of-function assays confirmed that KDM3A could regulate CMEC pyroptosis by regulating the PI3K/Akt signaling pathway.

### 3.7. KDM3A Knockout Exacerbated I/R-Induced Cardiac Function Deterioration and Reduced the Perfusion of Coronary Microcirculation

**Figure 7:**
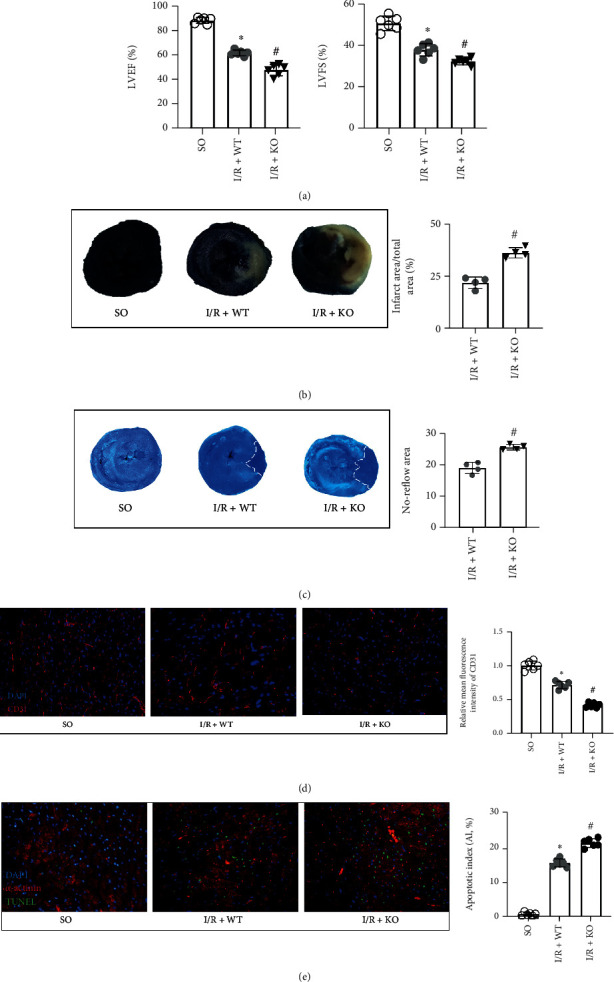
KDM3A knockout further exacerbates the deteriorated cardiac function and myocardial injury induced by I/R injury as well as reduces the perfusion of coronary microcirculation. (a) The changes in cardiac function, including LVEF and LVFS, were evaluated by echocardiography in rats of different groups (*n* = 6, ^∗^*p* < 0.05, as compared to the SO group; ^#^*p* < 0.05, as compared to the H/R+WT group). (b) Representative images of the myocardial infarction area of each group, and the ratio of the infarction area to the total left ventricular area was calculated (*n* = 4,  ^∗^*p* < 0.05, as compared to the SO group; ^#^*p* < 0.05, as compared to the H/R+WT group). (c) Representative images of thioflavin-S and Evan's blue dual-staining were performed to detect the no-reflow area after I/R injury of each group (*n* = 4,  ^∗^*p* < 0.05, as compared to the SO group; ^#^*p* < 0.05, as compared to the H/R+WT group). (d) Representative images of CD31 fluorescence staining were conducted to estimate the capillary density. Scale bar = 20 *μ*m. (e) Representative images and averaged data of the apoptotic index in rat heart tissue of each group. TUNEL staining (green) indicates apoptotic nuclei; DAPI counterstaining (blue) indicates total nuclei. Scale bar = 20 *μ*m. The apoptotic index (AI) is presented as the percentage of TUNEL-positive nuclei to the total number of nuclei (*n* = 6,  ^∗^*p* < 0.05, as compared to the SO group; ^#^*p* < 0.05, as compared to the H/R+WT group).

We aimed to validate our *in vitro* findings *in vivo* using wild-type and KDM3A gene globe-knockout rats. Echocardiography was performed to evaluate cardiac functions for each group. In Figure 7(a) and Supplementary Figure 2, KDM3A knockout reduced the percentage of LVEF and LVFS, compared to the WT group in response to I/R injury. Besides, KDM3A gene deletion enlarged the myocardial infarct sizes (Figure 7(b)). In addition, I/R injury significantly increased the no-reflow area, accompanied by a marked decrease in capillary density. However, the situation is getting worse as manifested by an enlarged no-reflow area and reduced fluorescence intensities of CD31 in the KDM3A-KO+I/R group (Figures 7(c) and 7(d)). Moreover, the I/R insult resulted in massive cell death in the myocardial tissue, which was further exacerbated by KDM3A knockout. TUNEL-positive cells were increased in the KDM3A-KO+I/R group, relative to the KDM3A-WT+I/R group (Figure 7(e)). Thus, the evidence above indicates that KDM3A alleviates I/R injury by remediating coronary microcirculation dysfunction.

## 4. Discussion

Although KDM3A protects cardiomyocytes from I/R injury, its involvement and potential molecular mechanisms in alleviating CMEC I/R injury have yet to be fully established. To elucidate the cause-effect relationship between KDM3A and CMEC I/R injury, we created CMEC I/R injury models and tried to decipher its significance. The KDM3A levels in CMECs were markedly reduced in response to H/R. KDM3A knockdown further impaired the proliferation, migration, and angiogenesis capacities of CMECs after H/R treatment. Besides, KDM3A deletion exacerbated H/R-induced inflammatory responses in CMECs and cell death, accompanied by marked upregulations of pyroptosis-associated cytokines. However, these negative outcomes were reversed by KDM3A upregulation. Notably, pretreatment with LY294002, a PI3K/Akt pathway inhibitor, partially reduced the beneficial effects of KDM3A upregulation. *In vivo*, KDM3A knockout exacerbated cardiac functions as well as myocardial damage and reduced the density of the capillary network during I/R injury. This is the first study to establish that KDM3A inhibits CMEC I/R injury, supporting the notion that KDM3A can mitigate I/R-induced CMEC pyroptosis via PI3K/Akt signaling pathway activation.

Fresh blood supply to ischemic tissues is the most efficient way of reducing ischemia-induced cardiomyocyte damage or death [3]. Nevertheless, microvascular I/R injury occurs in about 50% of patients, including those with successful revascularization treatment [33]. Despite blood flow of the epicardial artery being restored rapidly and effectively if microvascular I/R injury occurs, capillaries that nourish cardiomyocytes are ineffectively infused [11, 12]. This phenomenon emerges within several minutes after revascularization and lasts for a few weeks. Epidemiologically, microvascular injury is an independent predictive risk factor for in-hospital mortality and one-year major adverse cardiovascular events [34]. There is an intimate physiological association between CMECs and cardiomyocytes. Anatomically, each cardiomyocyte is surrounded by 3–4 CMECs [35]. The microcirculatory network formed by CMECs is far from merely being the interface between epicardial vessels and cardiomyocytes and also provides oxygen and nutrition, which are necessary for cardiomyocytes to survive. Moreover, under physiological conditions, CMECs are involved in cardiac metabolism, contractile performance, growth, and rhythmicity [14, 36, 37]. It has been reported that CMECs are the first responder cells to cardiac I/R injury, and their malfunctions exacerbate the impairment of cardiomyocytes, acting as dynamic sensors and modulators for myocardial functions [13, 38]. Therefore, there is a need to decipher the underlying mechanisms in microvascular I/R injury and to determine effective therapeutic targets for the efficient amelioration of the no-reflow phenomenon.

As a newly recognized pattern of inflammation-associated cell death, pyroptosis is implicated in cardiovascular disease development, especially atherosclerosis, AMI, and myocardial I/R injuries [20, 39, 40]. In stressful environments, myocardial reperfusion injury-induced danger-associated molecular patterns activate the NLRP3 inflammasome, which is a representative multimeric protein complex that acts as the innate immune signal receptor to sense multiple stimuli and induce inflammatory responses under pathological and physiological states. The specific pyroptotic caspase-1 is recruited to the NLRP3 inflammasome and converted into its active form via autocatalysis. Precursors of IL-1*β* as well as IL-18 are cleaved into their mature form by activated caspase-1, which also separates the C- and N-terminals of GSDMD. Consequently, numerous nanopore holes are formed on the cell membrane, leading to elevated secretion of proinflammatory cytokines and induction of secondary inflammation cascades, resulting in cell swell, lysis, and pyroptotic death [40–42]. Inhibiting NLRP3 inflammasome activation in the early reperfusion period can ameliorate AMI-induced deterioration of cardiac performance and minimize the infarct size [43]. Besides, Bai et al. reported on intimate interactions between endothelial dysfunctions and NLRP3 inflammasome-associated pathways. Moreover, they highlighted the significance of the NLRP3 inflammasome in endothelial dysfunction-associated inflammatory diseases [23]. Sun et al. reported that myocardial I/R injury provoked the canonical NLRP3/caspase-1-depended pyroptosis pathway. However, gastrodin treatment inhibited CMEC pyroptosis and proinflammatory cascades, reducing myocardial infarct size, alleviating inflammatory cell infiltration, and increasing capillary formation [24]. Consistently, our findings indicated that the proliferation, migration, angiogenesis, and secretory capacities of CMECs after H/R treatment were significantly compromised, accompanied by upregulations of the NLRP3/caspase-1 signaling pathway-related inflammatory molecules and augmentation of CMEC pyroptosis. Findings from the subsequent *in vivo* experiment affirmed that I/R injury-induced CMEC pyroptosis exacerbates cardiac malfunction and maladaptive remodeling. Therefore, the development of novel preventive methods and effective treatment strategies to inhibit CMEC pyroptosis is a potential therapeutic approach for ameliorating myocardial I/R injury.

Phosphoinositide 3-kinases (PI3K)/serine/threonine kinase (Akt) is a ubiquitous signaling pathway in multiple cell biological processes and promotes cell survival [44]. Akt is a key downstream mediator of PI3K, and its activation form (phosphorylated Akt, p-Akt) can interact with various important regulatory factors that mediate different pathological processes, including inflammation, apoptosis, and oxidative stress [44, 45]. The PI3K/Akt pathway is a vital mediator in protecting diverse types of cells from pyroptosis during I/R injury. Diao et al. documented that hypothermia treatment protects hippocampal neurons against neuronal I/R-mediated pyroptosis by activating the PI3K/Akt pathway [46]. They also found that treatment with a specific NLRP3 inflammasome inhibitor, which is the downstream element of PI3K/Akt, results in comparable protective effects on cellular viability. Li et al. reported that docosahexaenoic acid (DHA) ameliorates liver ischemia-reperfusion injury by downregulating the levels of pyroptosis-related proteins (NLRP3 as well as cleaved caspase-1) and reducing the secretion of proinflammatory cytokines. However, incubation with LY294002 significantly abolished these beneficial effects [45]. In rat myocardial I/R injury models, Xu et al. unravelled that Aesculin attenuated reperfusion arrhythmias and myocardial damage, improved the hemodynamic functions, and remitted the inflammatory responses as well as cardiomyocyte pyroptosis. However, Aesculin-mediated cardioprotective and NLRP3 inflammasome suppression effects were blocked by the Akt inhibitor [47]. In this study, compared to the enhanced CMEC pyroptosis and upregulation of NLRP3/caspase-1 signaling pathway-related molecules, expressions of p-Akt were significantly suppressed in CMEC H/R injury models. Therefore, the PI3K/Akt signaling pathway might be a vital mediator of CMEC pyroptosis and a potential candidate for the regulation of CMEC I/R injury.

Our findings inform on the development of protective approaches against myocardial I/R injury in a KDM3A-dependent ETS1 enhancement through impacting multipathogenetic episodes including apoptosis, inflammation, and ROS [26]. However, it has not been established whether and how KDM3A attenuates CMEC I/R injury. As a histone demethylase, the biochemical features of KDM3A have been characterized, but its physiological role in different diseases has yet to be conclusively determined. KDM3A was originally found to be elevated and had a vital role in hypoxia [48]. Studies on KDM3A have majorly focused on cancer, where it enhances tumor cell proliferation, migration, invasion, and angiogenesis. Therefore, KDM3A is regarded as a poor prognostic marker [27, 28]. In recent years, epigenetic modulations were considered to be a crucial factor in the pathogenesis of cardiovascular diseases, and the functions of KDM3A have been explored [29, 30]. In our unpublished study of cardiac metabolic memory injury under diabetic conditions, KDM3A was found to be an upstream modulator of NF-*κ*B/p65 and a regulator of cardiomyocyte injury by disturbing apoptosis, inflammation, and ROS. Our findings confirm the protective roles of KDM3A against AMI-induced myocardial injury and maladaptive cardiac remodeling [30]. KDM3A is also involved in cardiac hypertrophy as well as fibrosis [31] and is a crucial upstream regulator of the PI3K/Akt signaling pathway [31, 49]. We assessed the significance and mechanisms of KDM3A in CMEC I/R injury in the present study. It was established that KDM3A upregulation attenuated H/R-induced CMEC malfunctions, accompanied by a marked increase in p-Akt levels. However, expressions of cleaved caspase-1, IL-1*β*, NLRP3, and IL-18 were remarkably downregulated. On the contrary, KDM3A knockout exacerbated these abnormalities. However, LY294002 partially blocked the beneficial effects of KDM3A overexpression. *In vivo*, KDM3A knockout aggravated cardiac dysfunction, increased the infarct size, reduced capillary density, and enhanced pyroptosis-associated protein expressions by inactivating the PI3K/Akt signaling pathway.

In summary, KDM3A protects CMECs from H/R injury-induced pyroptosis via PI3K/Akt signaling pathway activation. Therefore, interventional strategies that selectively regulate KDM3A are potential candidates for mitigation of I/R injury. Future studies should investigate the roles of endogenous upstream targets of KDM3A, including specific microRNAs, lncRNAs, and/or circRNAs in CMECs, by alternatively regulating KDM3A expressions, which may also represent a novel modality for protecting CMECs against I/R injury.

## Data Availability

The original contributions presented in the study are included in the article; further inquiries can be directed to the corresponding author upon reasonable request.

## Supplementary Materials

Supplementary Materials Supplementary Figure 1: KDM3A deletion exerts no obvious influence on CMECs' function under normoxic conditions. Supplementary Figure 2: echo raw data and time point zero heart function of each group. Supplementary Figure 3: uncropped blot corresponding to Figures 2(a) and 2(b). Supplementary Figure 4: uncropped blot corresponding to Figures 4(a) and 4(b). Supplementary Figure 5: uncropped blot corresponding to Figures 6(a) and 6(b).
