# Supplementary material for: KDM3A Attenuates Myocardial Ischemic and Reperfusion Injury by Ameliorating Cardiac Microvascular Endothelial Cell Pyroptosis
Source: Oxid Med Cell Longev. 2022 Sep 2;2022:4622520. doi: 10.1155/2022/4622520 (PMC9463006; doi:10.1155/2022/4622520)

Supplementary Figures

Supplementary Figure 1.


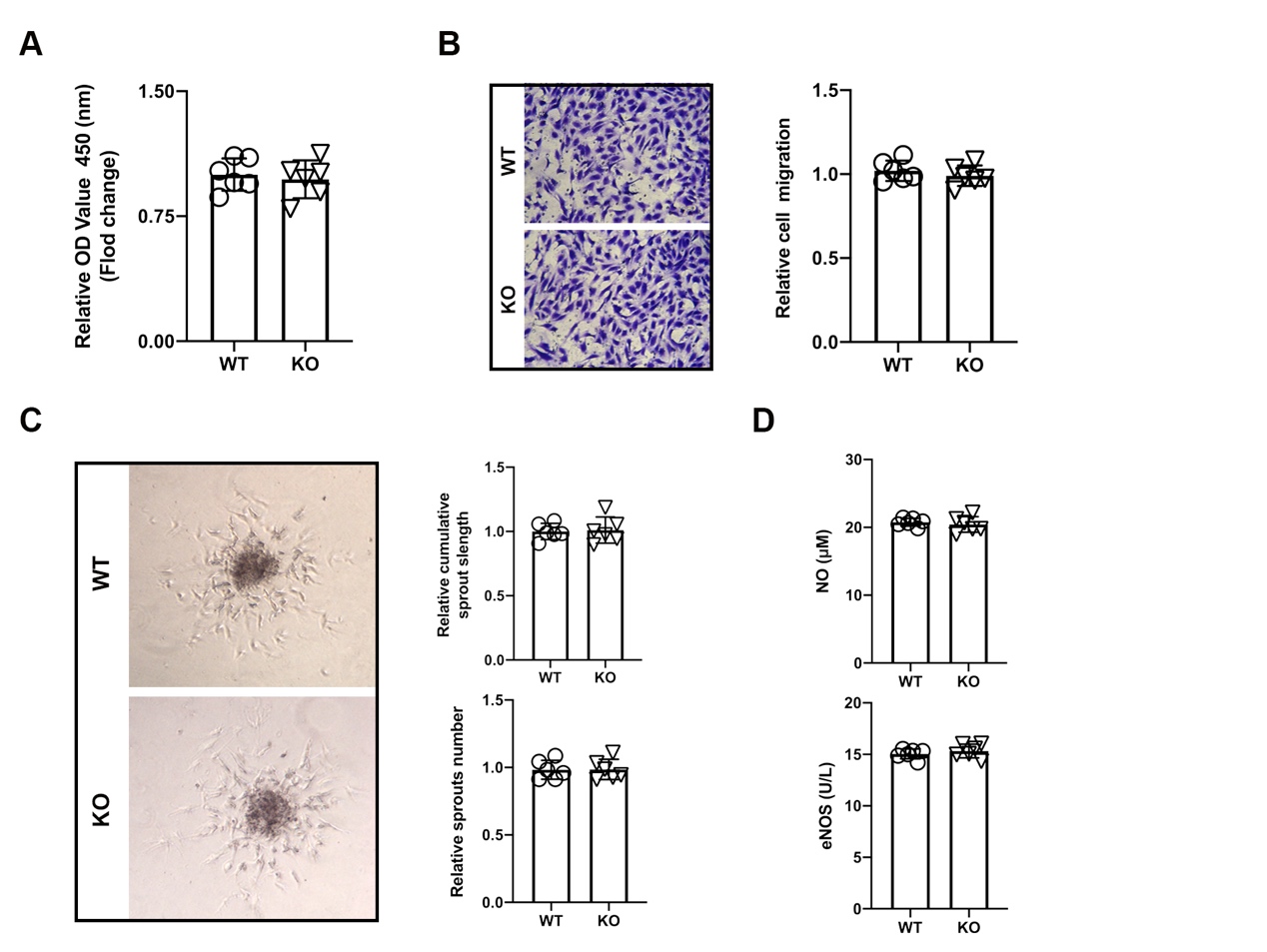


**KDM3A deletion exerts no obvious influence on CMECs' function under normoxic conditions.** (A) The proliferation capability of WT and KDM3A-KO CMECs was detected by the CCK-8 assay. (B) The migration capability of WT and KDM3A-KO CMECs was measured by the transwell chamber assay. (C) The neovascularization capacity of WT and KDM3A-KO CMECs was measured by the sprouting assay. (D) The levels of eNOS and NO in the supernatant of WT and KDM3A-KO groups were measured. (n=6)

Supplementary Figure 2.


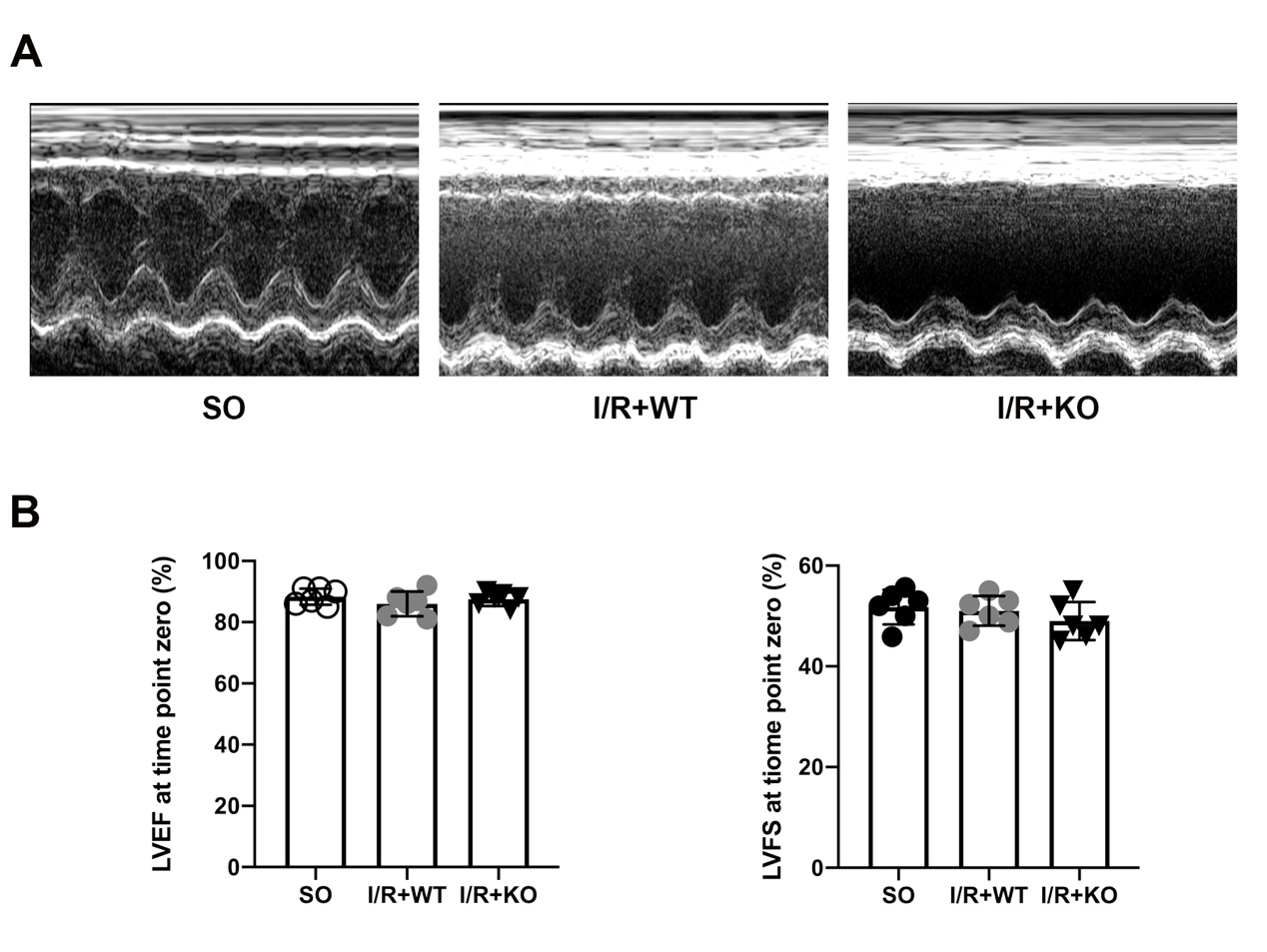


**Echo raw data and Time points zero heart function of each group. A.** Representative ultrasound images of each group after 24 h reperfusion. **B.** Heart function at time point zero of each group (n=6).

Supplementary Figure 3.

Uncropped blot corresponding to Figure 2A and Figure 2B


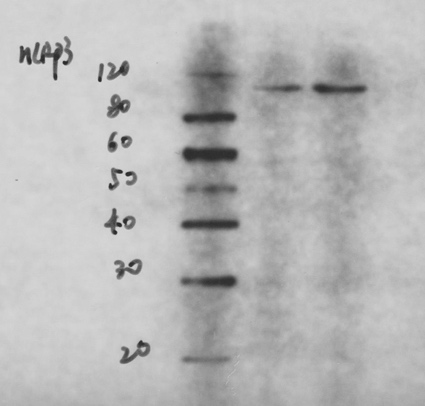

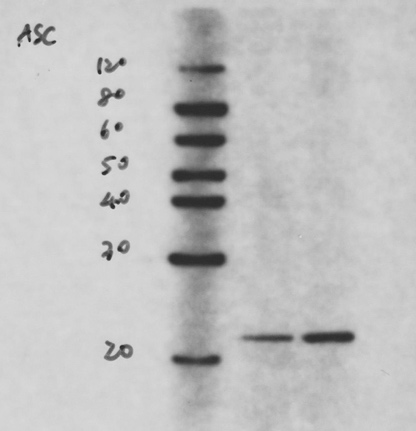

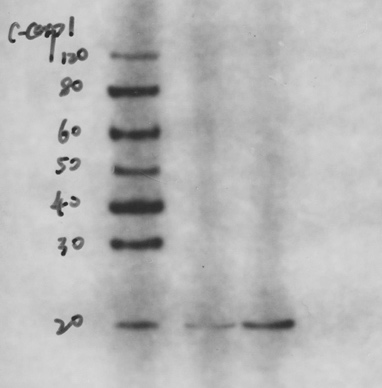


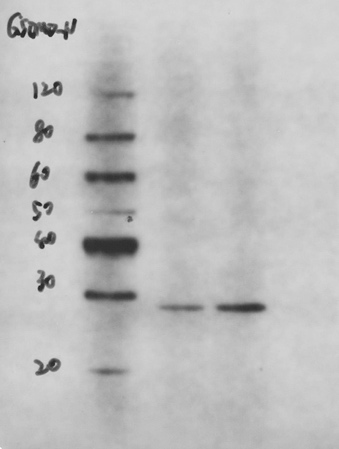

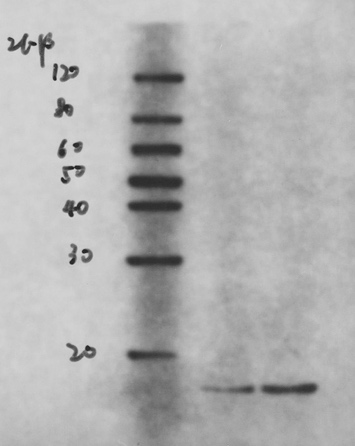

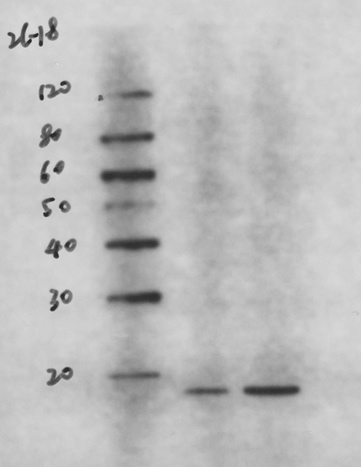


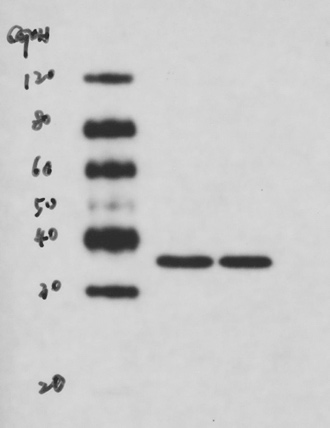

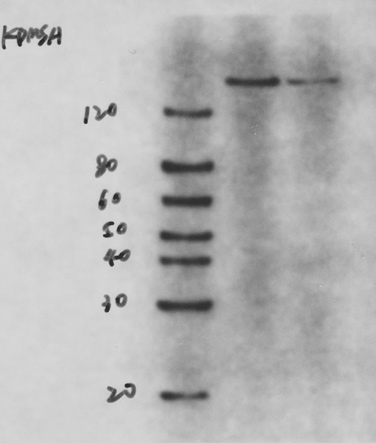

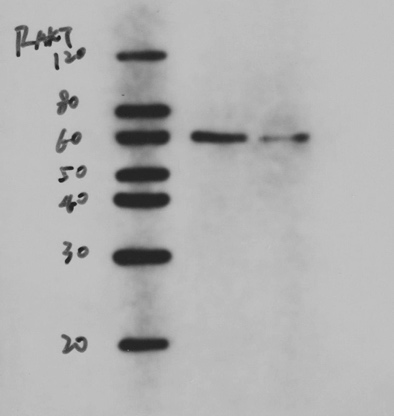

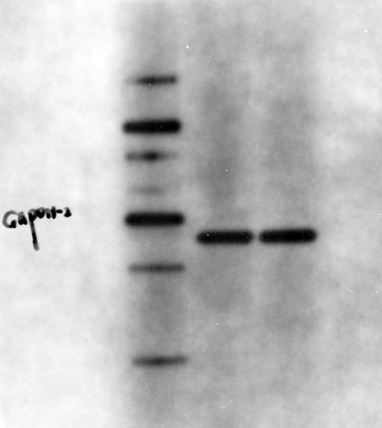


Supplementary Figure 4.

Uncropped blot corresponding to Figure 4A and Figure 4B


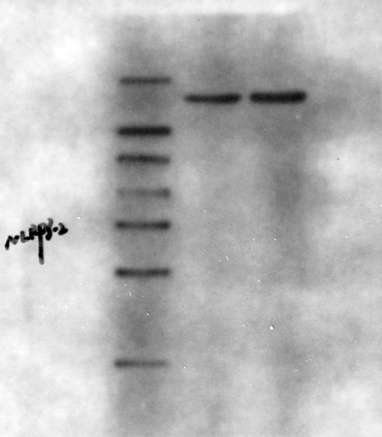

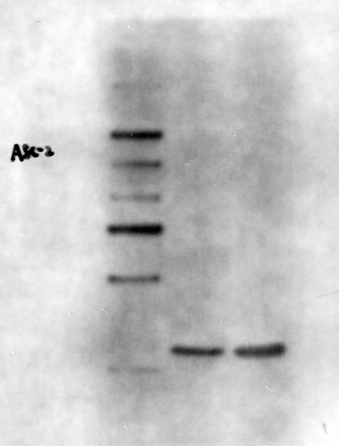

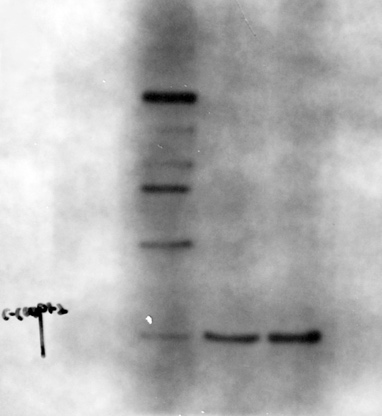


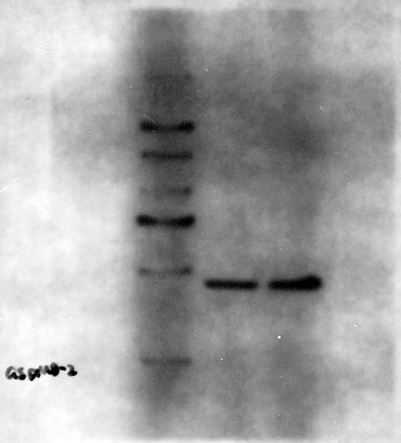

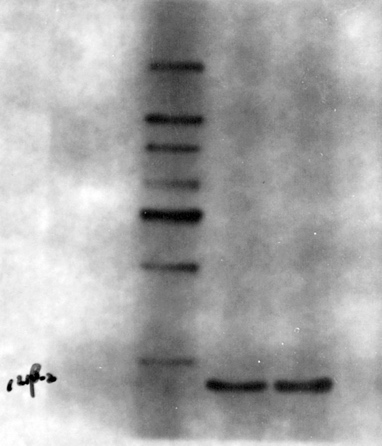

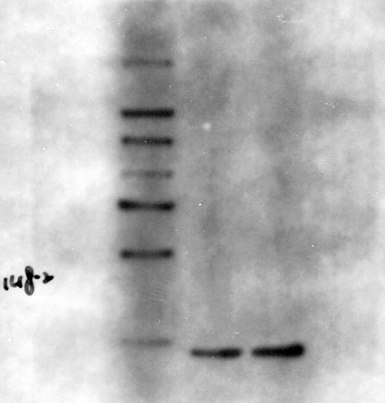


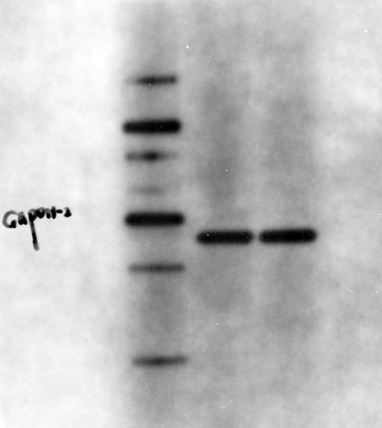

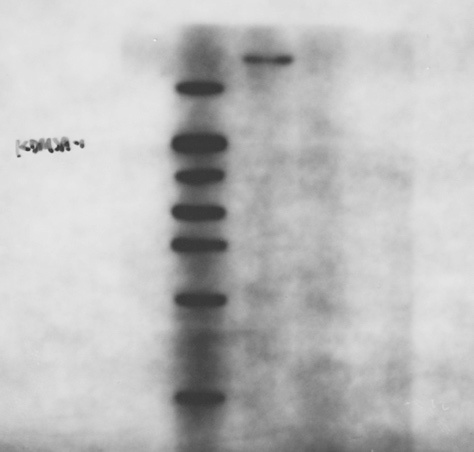

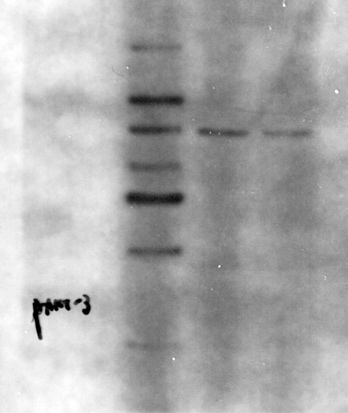

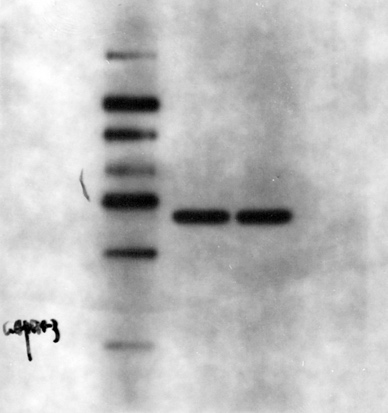


Supplementary Figure 5.

Uncropped blot corresponding to Figure 6A and Figure 6B


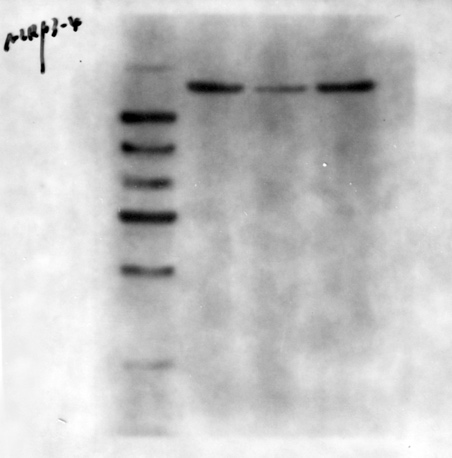

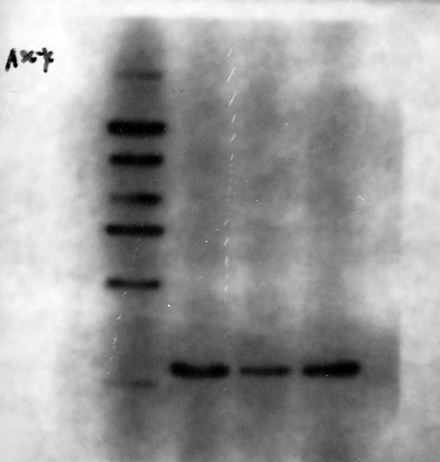

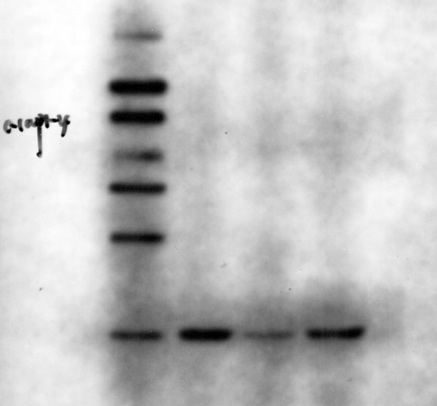

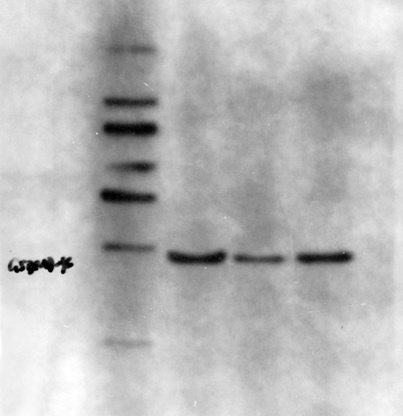

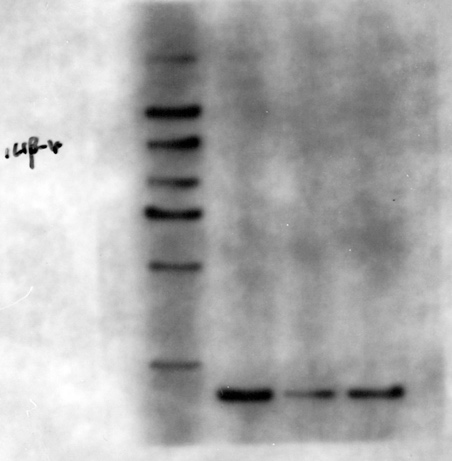

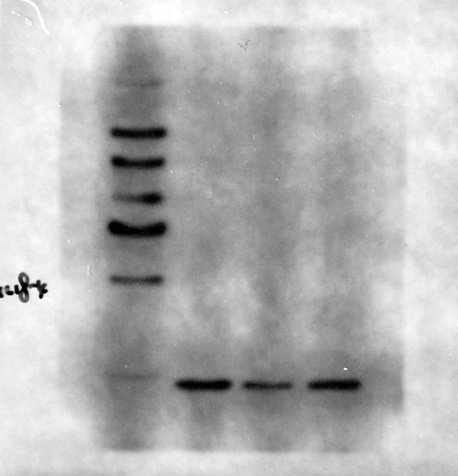

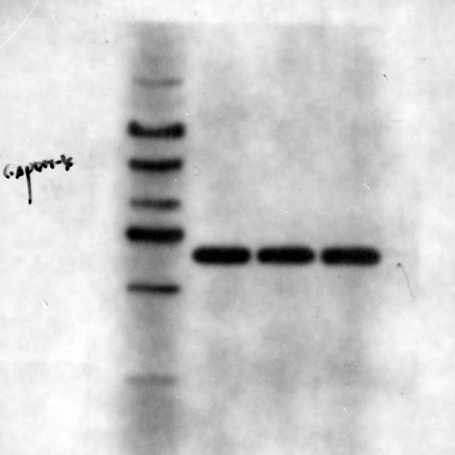

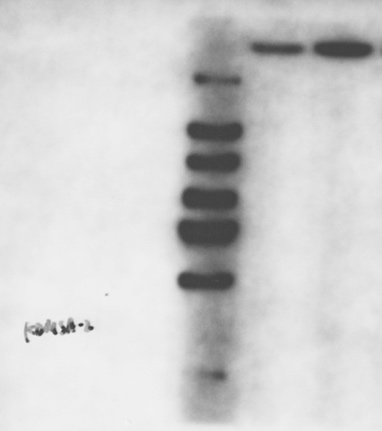


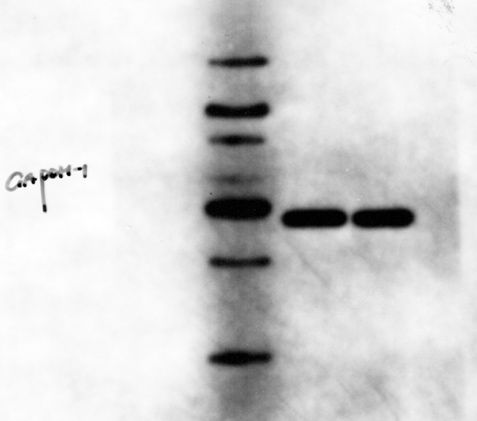

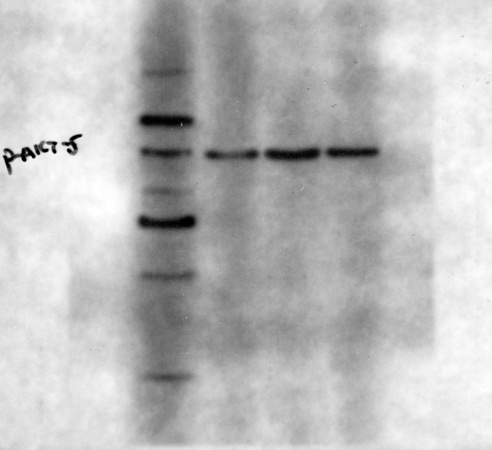

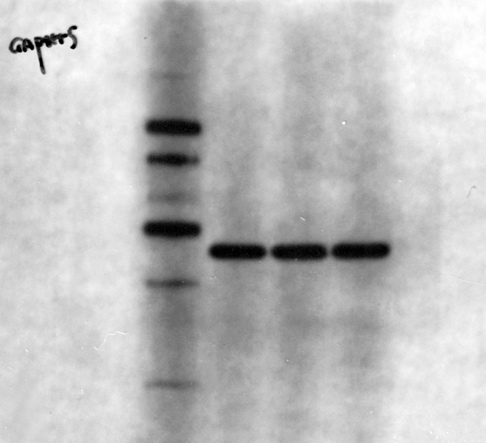

Supplement: Supplementary Materials — Supplementary Figure 1: KDM3A deletion exerts no obvious influence on CMECs' function under normoxic conditions. Supplementary Figure 2: echo raw data and time point zero heart function of each group. Supplementary Figure 3: uncropped blot corresponding to Figures 2(a) and 2(b). Supplementary Figure 4: uncropped blot corresponding to Figures 4(a) and 4(b). Supplementary Figure 5: uncropped blot corresponding to Figures 6(a) and 6(b). [file 4622520.f1.docx]
